# One-Step Synthesis of Nitrogen-Doped Hydrophilic Mesoporous Carbons from Chitosan-Based Triconstituent System for Drug Release

**DOI:** 10.1186/s11671-019-3075-y

**Published:** 2019-07-30

**Authors:** Xianshu Wang, Hongyan Pan, Qian Lin, Hong Wu, Shuangzhu Jia, Yongyong Shi

**Affiliations:** 1 School of Chemistry and Chemical Engineering, Guizhou University, Guiyang, 550025 Guizhou People’s Republic of China; 2 Key Laboratory of Green Chemical and Clean Energy Technology, Guiyang, 550025 Guizhou People’s Republic of China; 3 School of Pharmacy, Guizhou University of Traditional Chinese Medicine, Guiyang, 550025 Guizhou People’s Republic of China

**Keywords:** Chitosan, Nitrogen-doped mesoporous carbon spheres, Hydrophilicity, Drug delivery carrier, Hydroxycamptothecin

## Abstract

In situ nitrogen-doped hydrophilic mesoporous carbon spheres with different carbon-to-silicon (C/Si) ratios (NMCs-*x*/3, *x* = 5, 6, 7, and 8) were prepared by one-step method coupled with a spray drying and carbonizing technique, in which triblock copolymer (F127) and tetraethyl orthosilicate (TEOS) were used as template agents, and biocompatible chitosan (CS) was used as the carbon source and nitrogen source. These carbon materials were characterized by TG, BET, XRD, Raman, FTIR, TEM, XPS, and contact angle measuring device. The adsorption and release properties of mesoporous carbon materials for the poorly soluble antitumor drug hydroxycamptothecin (HCPT) were investigated. Results showed that nanospherical mesoporous carbon materials were successfully prepared with high specific surface area (2061.6 m^2^/g), narrowly pore size distribution (2.01–3.65 nm), and high nitrogen content (4.75–6.04%). Those NMCs-*x* showed a satisfactory hydrophilicity, which gradually increased with the increasing of surface N content. And the better hydrophilicity of NMCs-*x* was, the larger adsorption capacity for HCPT. The absorption capacity of NMCs-*x* towards HCPT was in the following orders: *q*_NMCs-5/3_ *> q*_NMCs-6/3_ *> q*_NMCs-7/3_ *> q*_NMCs-8/3_. NMCs-5/3 had the largest saturated adsorption capacity of HCPT (1013.51 mg g^−1^) and higher dissolution rate (93.75%).

## Introduction

Mesoporous silica [1, 2], mesoporous molecular sieves [3], mesoporous carbon [4–6], and other materials have been widely used in the field of biomedicine. Among them, mesoporous carbon materials have better properties in terms of specific surface area, pore volume, good chemical stability, and thermal stability [7], hence are more suitable to serve as an excellent drug loading materials. It has been reported that mesoporous carbon materials have been extensively used in antitumor drug loading (camptothecin [8], doxorubicin [9–12], paclitaxel [13–16], photothermal therapy, integrative therapy, labeling of fluorescent cells, biosorption of human toxic substances, medical imaging, and biosensing [17].

Currently, phenolic resins [18, 19] and sucrose [20, 21] are usually used as carbon sources to prepare mesoporous carbon. However, there are potential environment hazards associated with phenolic resins used as a carbon source. Also, sucrose has the disadvantages of the complex preparation process and high cost. The hydrophilicity of mesoporous carbon materials prepared from these two carbon source materials is poor, which constrains the use of mesoporous carbon as drug loader in injection and blood circulation [17]. In order to increase the hydrophilicity of mesoporous carbon, many approaches have been proposed to modify the mesoporous carbon by mixed acid oxidation [9, 12, 22] or directly doped nitrogen on mesoporous carbon materials [23–25]. However, strong oxidation could lead to a negative effect on the surface properties and pore structure of the mesoporous carbon, affecting their drug loading potential. On the other hand, nitrogen doping after treatment is cumbersome and costly, which is not acceptable for mass production.

Chitosan is a kind of biomass with abundant carbon content, and hydroxyl (–OH) and amino(–NH_2_) [26, 27]. Mesoporous carbon materials are prepared by using chitosan as carbon source.

Currently, chitosan has been reported as a carbon source to prepare mesoporous carbon by evaporation-induced self-assembly (EISA) method. For example, Sun [28] prepared mesoporous carbon with a pore size of 2–16 nm and specific surface area of 293–927 m^2^/g using chitosan-protic salt as carbon source and F127 as a template. Feng [29] prepared mesoporous carbon with a pore size of 5–15 nm and specific surface area of 41–457 m^2^/g using chitosan as carbon source and F127 as a template. Andrzej [30] prepared mesoporous carbon with a pore size of 3–20 nm and specific surface area of 600–1337 m^2^/g using chitosan as carbon source and colloidal SiO_2_ as a template. However, these prepared mesoporous carbon materials are featured with a wider pore size distribution, lower specific surface area, irregular morphology, and larger particle size (> 1 μm). The molecular sizes of common anticancer drugs are usually in the range of 1.1–1.9 nm, such as paclitaxel, doxorubicin, and hydroxycamptothecin (HCPT), which are 1.90 nm × 1.19 nm × 0.07 nm, 1.52 nm × 1.08 nm × 0.71 nm, 1.14 nm × 0.69 nm × 0.44 nm, respectively (calculated by Materials Studio software). Generally speaking, the narrow pore size distribution of porous materials is beneficial to the mass transfer of adsorbate molecules, and the suitable pore size of porous materials is 1.5~3.0 times of adsorbate molecules size [31]. Thus, the mesoporous carbon materials as a drug carrier should have a narrow range in pore size with large volume, high specific surface area, good biocompatibility and hydrophilicity, and the nanospherical morphology. The spherical mesoporous carbon with a diameter less than 1 μm was prepared by spray drying technology in our previous reports [32]. However, though the prepared mesoporous carbon material showed higher hydrophilicity (contact angle theta is 124.1^o^) than that of the sample prepared with sucrose as carbon source (contact angle theta is 161.9^o^), the hydrophilicity and the specific surface area of mesoporous carbon is still unsatisfactory due to the less amount of oxygen-containing groups and the serious shrinkage and collapse of the organic skeleton formed by mesoporous carbon precursors during carbonization. It has been reported that hydrolytic polycondensation of tetraethyl orthosilicate (TEOS) in acidic solution can produce silicic acid aggregates with rich silicon hydroxyl groups connecting with ether bond of hydrophilic segment of F127 by hydrogen bonding [33], which can prevent the shrinkage and collapse of carbon structure during carbonization [18] and increase the oxygen-containing groups of mesoporous carbon materials.

Herein, chitosan was used as carbon source and nitrogen source, and F127 and TEOS were used as templates to prepare hydrophilic nano-mesoporous carbon materials with spherical morphology by using spray drying coupled with carbonization technique. The effects of different carbon-to-silicon ratios (C/Si) on the pore structure, composition, and hydrophilic properties of NMCs were examined, and the adsorption and release properties of mesoporous carbon materials for the poorly soluble antitumor drug hydroxycamptothecin (HCPT) were investigated.

## Materials and Methods

### Raw Materials and Reagents

Amphiphilic triblock copolymer F127 (*M*_*w*_ = 12,600, EO_106_-PO_70_-EO_106_, Sigma-Aldrich, USA), TEOS (Aladdin Reagent Company, America), CS (degree of deacetylation ≥ 95%, viscosity 100~200 mPa s; Aladdin Reagent Company, America), HCPT (HCPT-160201; Chengdu Yuancheng Biotechnology Co., Ltd., China), and glacial acetic acid, hydrochloric acid, anhydrous ethanol, Tween-80, monopotassium phosphate, and sodium hydroxide (analytically pure; Shanghai Sinopharm Chemical Reagent Co., Ltd., China) were used. Deionized water was used in all experiments.

### Preparation of NMCs

**Fig 1:**
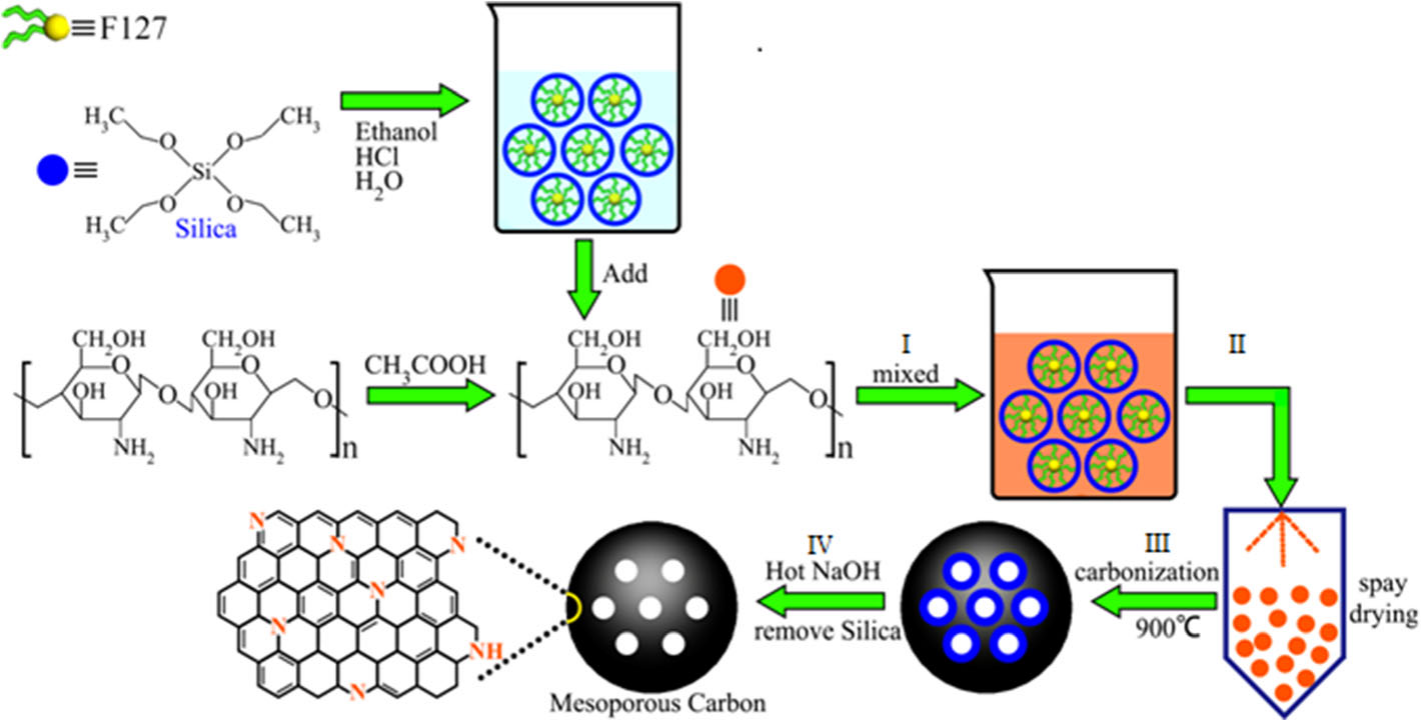
Schematic illustration of the fabrication processes of NMCs

The preparation of nitrogen-doped mesoporous carbon can be described by the synthesis schematic diagram in Fig. 1. There are four steps: (I) using chitosan as a carbon and nitrogen source, and triblock copolymer F127 and tetraethyl orthosilicate (TEOS) as template agents. In an alcohol-water biphasic system, spherical micelles were formed by double electron coupling between F127 and TEOS. Si–OH was then formed by hydrolysis, and –NH_2_ in CS formed hydrogen bonds in the acidic condition, leading to the creation of a triconstituent system subsequently polymerized and cross-linked to form a complex; (II) spray molding of the composite material was assembled by triconstituent system through spray drying process; (III) removal of F127 by roasting in an nitrogen atmosphere and carbonization; and (IV) thermal alkali removal of silicon to form mesoporous carbon materials. Four representative samples were prepared with a varying C/Si ratio and labeled as NMCs-5/3, NMCs-6/3, NMCs-7/3, and NMCs-8/3. A typical synthesis experiment involved the following steps: (a) preparation of CS solution—CS (7.0, 8.4, 9.8, or 11.2 g) was dissolved in a 5% acetic acid aqueous solution at 40 °C to prepare a 2.1% CS solution. (b) NMCs were prepared by dissolving 2.1 g of F127 in 50 mL of ethanol solution at 40 °C, followed by addition of 15.6 mL of TEOS and 0.2 M HCl (15 mL) for hydrolysis. After 10 min of reaction, the solution was transferred into the CS solution and mixed for 60 min. The mixture was then left to rest at room temperature for 60 min and then dried with a spray dryer (BUCHI B-290, BUCHI, Switzerland) at an inlet air temperature of 170 °C and feeding flow rate of 3.5 mL/min. The obtained sample was labeled as CS/SiO_2_/F127; and (c) carbonization process—the CS/SiO_2_/F127 powder was placed in a tube furnace, protected with the nitrogen flow rate of 200 cm^3^/h, and heated at a rate of 2 °C/min until 410 °C. This temperature was then held for 2 h, then increased at a rate of 5 °C/min until 900 °C, and calcined for 2 h to obtain the C–Si composite material. Si was removed from the obtained C–Si composite material using hot alkali with 1 M NaOH aqueous solution at 85 °C twice, washed with deionized water until it a neutral pH reading was obtained, and dried at 100 °C to yield the mesoporous carbons (Fig. 1). The obtained materials were labeled as NMCs-5/3, NMCs-6/3, NMCs-7/3, and NMCs-8/3 according to the amount of CS used in the solution (7.0, 8.4, 9.8, or 11.2 g CS, respectively).

### Method of Characterization

The specific surface area, pore volume, and pore size of mesoporous carbons were tested using a Micrometrics ASAP2020 N_2_ adsorption/desorption physical adsorption instrument. The samples were pre-degassed under vacuum conditions(76 mmHg) at 120 °C for 12 h. The specific surface area (*S*_BET_) was calculated using the Barrett–Emmer–Teller method, whereas the pore volume (*V*_BJH_) and pore size (*D*_BJH_) were calculated using the Barrett–Joyner–Halanda (BJH) model, in which the pore volume was calculated as the absorption capacity at relative pressure *P*/*P*_0_ = 0.975.

The elemental composition (C, H, O, N) of NMCs was characterized using an ElementarVario EL Type III elemental analyzer.

The pyrolysis process of F127, CS, and the ternary system spray intermediate product CS/SiO_2_/F127 was characterized using a Netzsch STA 449C Thermal Analyzer. The temperature range was set from room temperature to 1000 °C at a rate of 5 °C/min.

The crystal characteristics of NMCs were characterized using a Bruker D8 Advance X-ray diffractometer with CuKα radiation, incident wavelength *λ* of 0.154060 nm, at 40.0 kV and 40.0 mA, and a 2*θ* range of 0.9–4° (scanning speed: 0.5°/min, scanning step length 0.002°).

The morphology of mesoporous carbon was analyzed by a FEI Tecnai G2 F20 S-Twin transmission electron microscope at an accelerating voltage of 200 kV.

The atomic binding state of NMCs was characterized using a ThermoScienfticEscalab 250XI X-ray photoelectron spectrometer using a Al Kα radiation source and the following parameters: test energy, 1486.8 eV; test spot diameter, 500 μm; test tube voltage, 15 kV; tube current, 10 mA; ultimate pressure of analysis chamber, 2 × 10^–9^ mbar. Peak position correction was made according to C1s at 284.8 eV.

The water contact angle on the surface of the samples was tested using the Dataphysics OCA25 optical contact angle measuring device.

### Absorption of HCPT on NMCs

HCPT was accurately weighed to 10 mg and dissolved in 50 mL of absolute ethanol solution to prepare a 200 μg/mL of standard stock solution. Then, the stock solution was diluted to concentrations (0.4, 0.5, 1, 3, 5, 7, and 10 μg mL^–1^). An absolute ethanol solution was used as the reference solution to measure the absorbance value for each concentration of standard solutions by UV spectrophotometry at 385 nm. A regression analysis of mass concentration (C) with absorbance (A) was performed to obtain the regression equation *y* = 0.07573*x* + 0.04149; the standard curve had a good linear relationship between the absorbance and the concentration within the measured range of 0.4~10 μg/mL, with correlation coefficient *R*^2^ = 0.99947.

The loading of drugs into the NMCs was carried out with the method of organic solvent immersion solution. HCPT solution (0.2~1.2 mg mL^–1^) was prepared by dissolving a certain amount (6~36 mg) of HCPT in 30 mL absolute ethanol. Subsequently, 20 mg of the various NMCs were then added, mixed at 37 °C in a water bath for 24 h in the dark, and separated by centrifugation at 8000 r/min for 10 min. The supernatant was then extracted and the concentration of HCPT was detected by UV absorption spectroscopy at the maximum absorption wavelength of 385 nm. The drug carrier was placed in a vacuum and dry area at 40 °C for 24 h. The amount of drug adsorbed onto the NMC samples was determined according to the change of concentration before and after adsorption. The drug adsorption capacity for each NMC sample was calculated according to the following equation:

$$ \mathrm{Drug}\ \mathrm{adsorption}\ \mathrm{capacity}\left(\mathrm{mg}/\mathrm{g}\right)=\frac{\mathrm{Drug}\ \mathrm{content}\ \mathrm{in}\ \mathrm{NMCs}}{\mathrm{Amount}\ \mathrm{of}\ \mathrm{NMCs}} $$

### Drug Release of HCPT

Dynamic dialysis was used to detect drug dissolution from 15 mg of pure HCPT as well as from the drug-loaded NMCs (NMCs-5/3@HCPT, NMCs-6/3@HCPT, NMCs-7/3@HCPT, and NMCs-8/3@HCPT). An in vitro release test was performed in phosphate buffer solution (PBS) with pH 7.4 and pH 5.0 and 0.1% Tween-80 at 37 °C in the dark. The prepared PBS buffer-NMC sample solutions (pH 7.4 and pH 5.0) were placed in dialysis bags (MWCO = 14,000) and immersed in 500 mL of PBS at pH 7.4, pH 5.0, and stirred at 100 r/min at 37 °C. A 4-mL aliquot was retrieved at regular time intervals of 1, 2, 4, 6, 8, 10, and 12 h and replenished with fresh isothermal, isovolumetric PBS. The dialysate was extracted and centrifuged at 8000 r/min for 10 min; 1 mL of supernatant was extracted and diluted by 20X and its absorbance was measured by UV spectrophotometry at 385 nm. The drug concentration was calculated according to the standard curve, and the cumulative release of HCPT was calculated according to the following equation:

$$ Q\left(\%\right)=\frac{V_1{C}_n+{V}_2\sum {C}_{n-1}}{W}\times 100\% $$

where *V*_1_ is the medium volume (mL), *V*_2_ is the sampling volume (mL), *C*_*n*_ is the sample concentration of HCPT in the *n* times sampling, (μg mL^–1^), *n* is the number of sampling trials, and *W* is the drug content of HCPT in NMCs.

## Results and Discussion

### Determination of Carbonization Conditions

**Fig. 2:**
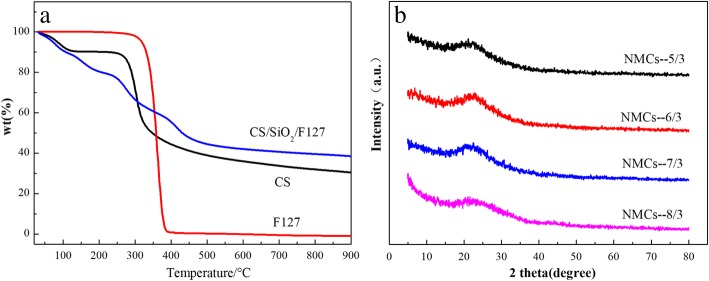
**a** TG curves of CS, F127, and CS/SiO_2_/F127. The heating rate was 5 °C/min under nitrogen flow. **b** XRD patterns of the NMCs of the NMCs-*x*/3(*x* = 5,6,7,8)

Figure 2a shows the thermogravimetric (TG) curves of free template agent F127, CS, and the CS/SiO_2_/F127 composite. It can be seen that F127 is almost completely pyrolyzed at 400 °C [34], with an approximately 99.6% weight loss, whereas CS experiences a 56% weight loss at 250–400 °C, followed by a plateau at 400–900 °C (1.53% weigh loss at 800–900 °C), suggesting that the carbon skeleton has formed at 800 °C. The weight loss of CS/SiO_2_/F127 occurred primarily below 500 °C (55.5%) mainly due to pyrolysis of F127 and CS; above 800 °C, the TG curves plateaued, suggesting that CS was almost completely carbonized. Andrzej [30] indicated that at high carbonization temperatures (1000–1100 °C), the nitrogen content of materials decreases. Therefore, the temperature of 400 °C was held for 2 h to remove F127, and the temperature of 900 °C was held for 3 h to ensure that the carbon materials had a higher nitrogen content and degree of graphitization.

### Effects of C/Si on Mesoporous Carbons

#### XRD Analysis of Mesoporous Carbons

The XRD spectra of the prepared samples NMCs-*x*/3(*x* = 5,6,7,8) is showed in Fig. 2b. There is a wide broad peak at 2*θ* = 23° on the four prepared samples, which is typical characteristic peaks of amorphous carbon materials [35]. It can be seen that the prepared carbon material NMCs-*x*/3 has an amorphous structure, which is consistent with the results reported in references [36, 37].

#### Pore Structure Analysis of Mesoporous Carbons

**Fig. 3:**
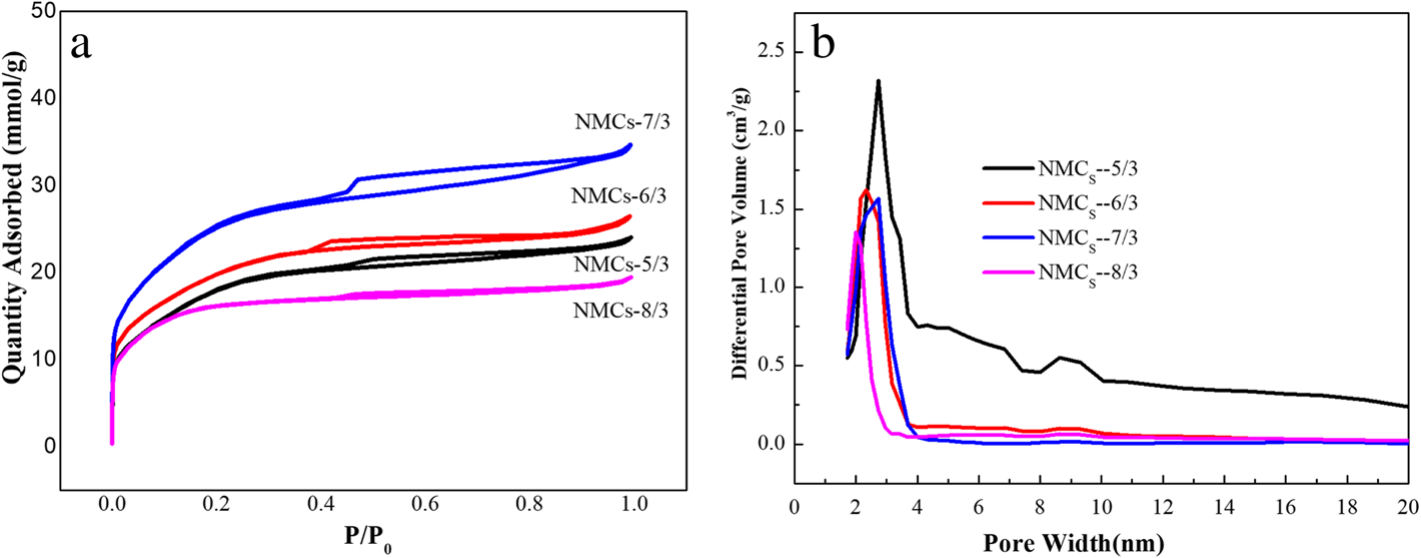
N_2_ adsorption-desorption isotherms (**a**) and pore size distribution curves (**b**) of NMC_S_

The N_2_ isothermal absorption and desorption curves of the four NMC samples and their pore size distribution curves are plotted (Fig. 3); the relevant pore structure data are provided in Table 1. After *P*/*P*_0_ ≥ 0.4, the N_2_ adsorption isotherms of the four samples show hysteresis loops typical of mesoporous carbon materials [38, 39]; from the four samples, the hysteresis loop for NMCs-7/3 is the largest (Fig. 3a). The pore size distribution graphs show that the pore size distribution of the carbon materials is relatively narrow, mainly within 2.01~3.65 nm (Fig. 3b), which is equivalent to 1.75~3.2 times of the aerodynamic equivalent diameter of HCPT. Kondo argued [31] that the smaller the pore size is, the slower the diffusion rate of the adsorbate into the pores is; the larger the pore size is, the adsorption potential of the adsorbate and the solid surface will be leading to poor absorption on the solid surface. Absorption capacity is the best when the pore size is 1.5~3.0 times of the aerodynamic equivalent diameter of the adsorbate. Thus, the pore size of the mesoporous carbons prepared herein is appropriate for the absorption of HCPT.

**Table 1:** Structural parameters of the nitrogen-containing mesoporous carbon materials

Sample name	*S*_BET_ (m^2^/g)	*V*_meso_ (cm^3^/g)	*D*_me_ (nm)
NMCs-5/3	1455.9	0.57	3.65
NMCs-6/3	1594.9	0.59	3.32
NMCs-7/3	2061.6	0.77	2.33
NMCs-8/3	1342.9	0.26	2.01

The pore structure data (Table 1) show that the mesoporous pore volume and BET-specific surface area of NMC-x materials increase then decrease with an increasing C/Si ratio, reaching a maximum value at C/Si = 7:3. This can be attributed to the following mechanism. At a low C/Si ratio (5/3), the amount of –OH and –NH_2_ on the CS is also small, whereas that of TEOS is relatively large; therefore, the amount of Si–OH formed by hydrolysis and polycondensation of TEOS is also large and therefore insufficient hydrogen bonding occurred with –OH and –NH_2_ on CS, leading to a reduction in the sols of the three-dimensional cross-linked network structure. Subsequently, following TEOS and F127 template removal, the mesoporous pore volume decreased. Moreover, since there is an excess of TEOS, the formed micelles are large and the average pore size obtained following TEOS removal is also large. Conversely, at a high CS content, and therefore high C/Si ratio (8/3), CS provided more –OH and –NH_2_, such that the Si–OH formed by the hydrolysis and polycondensation of TEOS is insufficient, leading to the formation of smaller and less micelles, decreasing the pore volume and pore size of mesoporous carbon. Evidently, at a C/Si ratio of 7:3, the available –OH and –NH_2_ groups are well matched with the amount of Si–OH on TEOS, leading to the formation of a larger mesoporous pore volume and BET-specific surface area.

#### TEM Analysis of Mesoporous Carbons

**Fig. 4:**
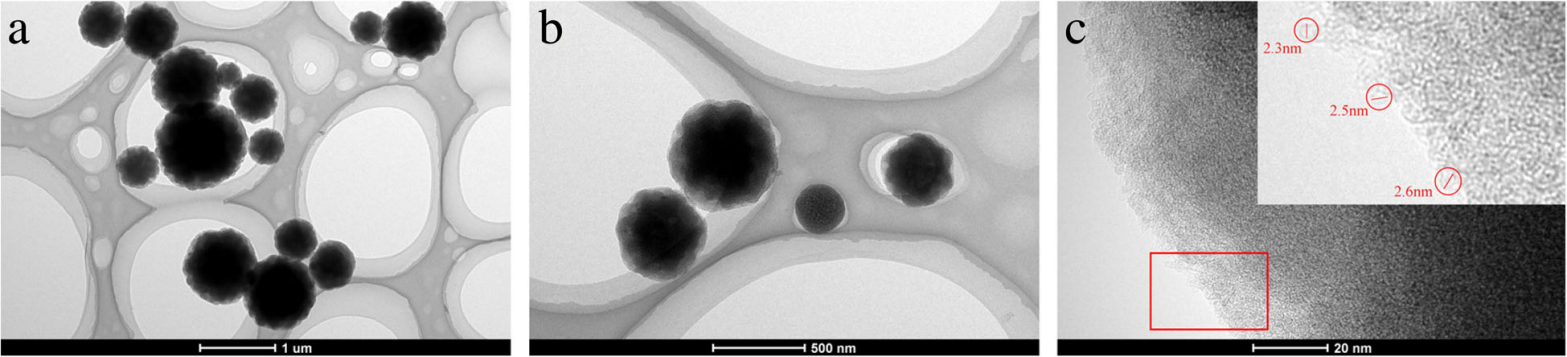
**a**–**c** TEM images of the NMCs-7/3 at different magnifications

Since NMC-7/3 has the largest specific surface area and mesopore volume, further tests on the pore distribution and microstructure are performed only for this sample, and the data are shown in Fig. 4. TEM images show that the prepared mesoporous carbon material NMC-7/3 has spherical structure at different magnifications and their particle sizes are all below 1 μm (Fig. 4a, b). The mesoporous carbon materials with a particle size of about 200 nm can efficiently carry drugs through cell membranes, thus exerting some unique therapeutic functions [40]. Figure 4c shows that the pore structure of the sample is visible and present a clear and typical worm-like structure [34] (Fig. 4c). It can be seen that ~2nm pore channels can be observed on the edge of the mesoporous carbon particles, which is generated by carbonization and reorganization of the chain structure of chitosan and removal of the template. However, the size of the small white dots observed in the surface of particles is generally less than 2 nm, which is due to the overlapping and intertwining of the chains formed by carbonization of chitosan.

### Composition and Hydrophilicity Analysis

#### Composition Analysis of NMCs

**Fig. 5:**
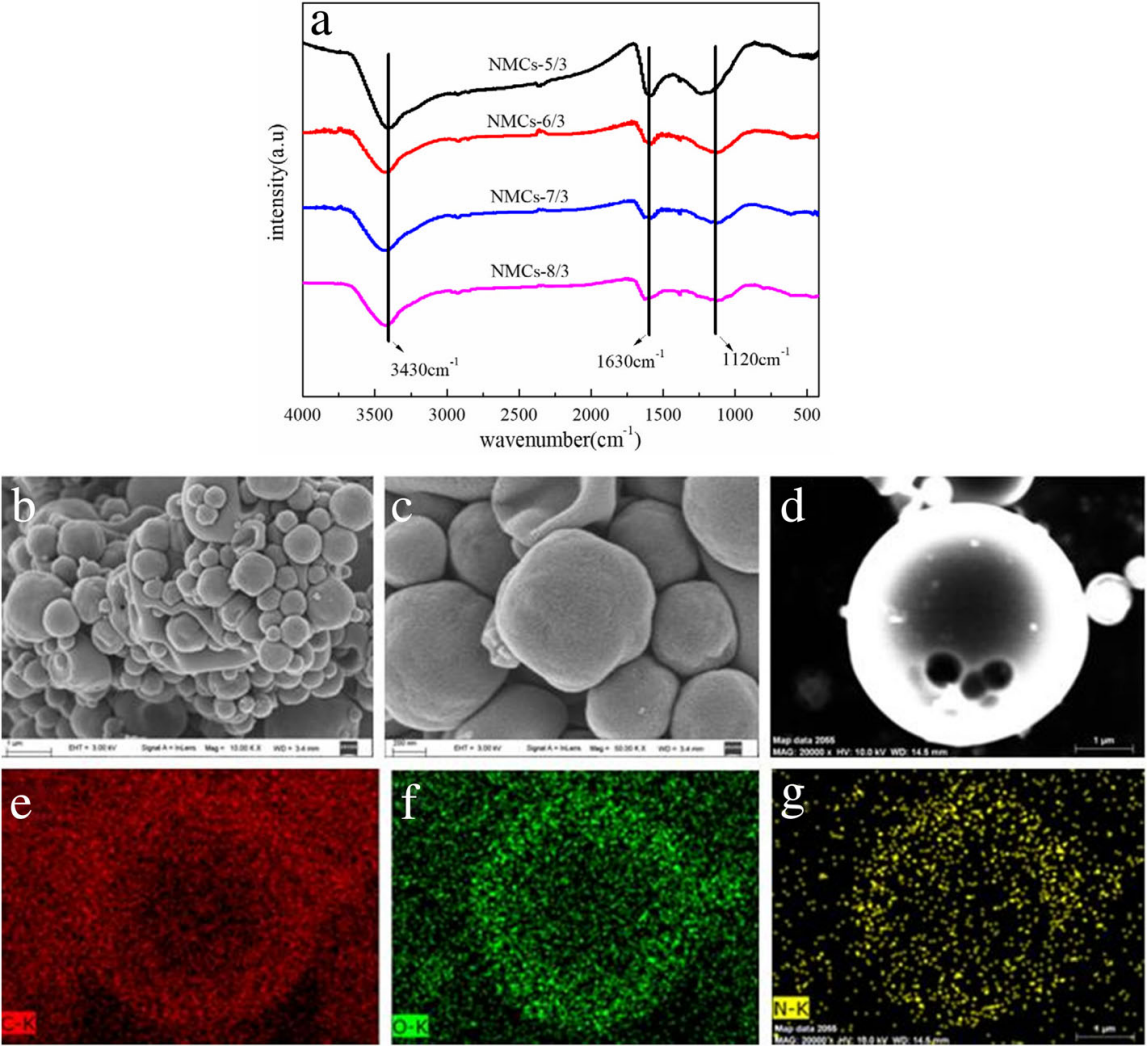
FTIR(**a**) patterns of the NMCs of the NMCs-*x*/3(*x* = 5,6,7,8); SEM image (**b**–**d**) of the sample NMC_S_-7/3 and elemental mapping (**e**–**g**) aimed at the sphere in inset picture of the SEM image (**d**),which responses to element C, O, and *N*, respectively.

FTIR spectrum of mesoporous carbon material NMCs-*x*/3 is shown in Fig. 5a; 3430 cm^−1^ is the stretching vibration absorption peak of N–H and O–H [41], 1630 cm^−1^ is the stretching vibration absorption peak of C=N and C=C, and 1120 cm^−1^ is the stretching vibration absorption peak of C–N and C–C, which indicates that nitrogen atoms are successfully incorporated into NMCs.

The SEM of NMC-7/3 (Fig. 5b–d) and the C(e), O(f), and N(g) elemental analysis of its surface (Fig. 5e–g) clearly show that the mesoporous carbon material prepared is spherical, but its size is not uniform. This is due to the spray drying process. The elements scanning data indicate that C, O, and N elements are distributed within the nanosphere mesoporous carbon. Therefore, *N* was successfully doped into the NMCs.

**Fig. 6:**
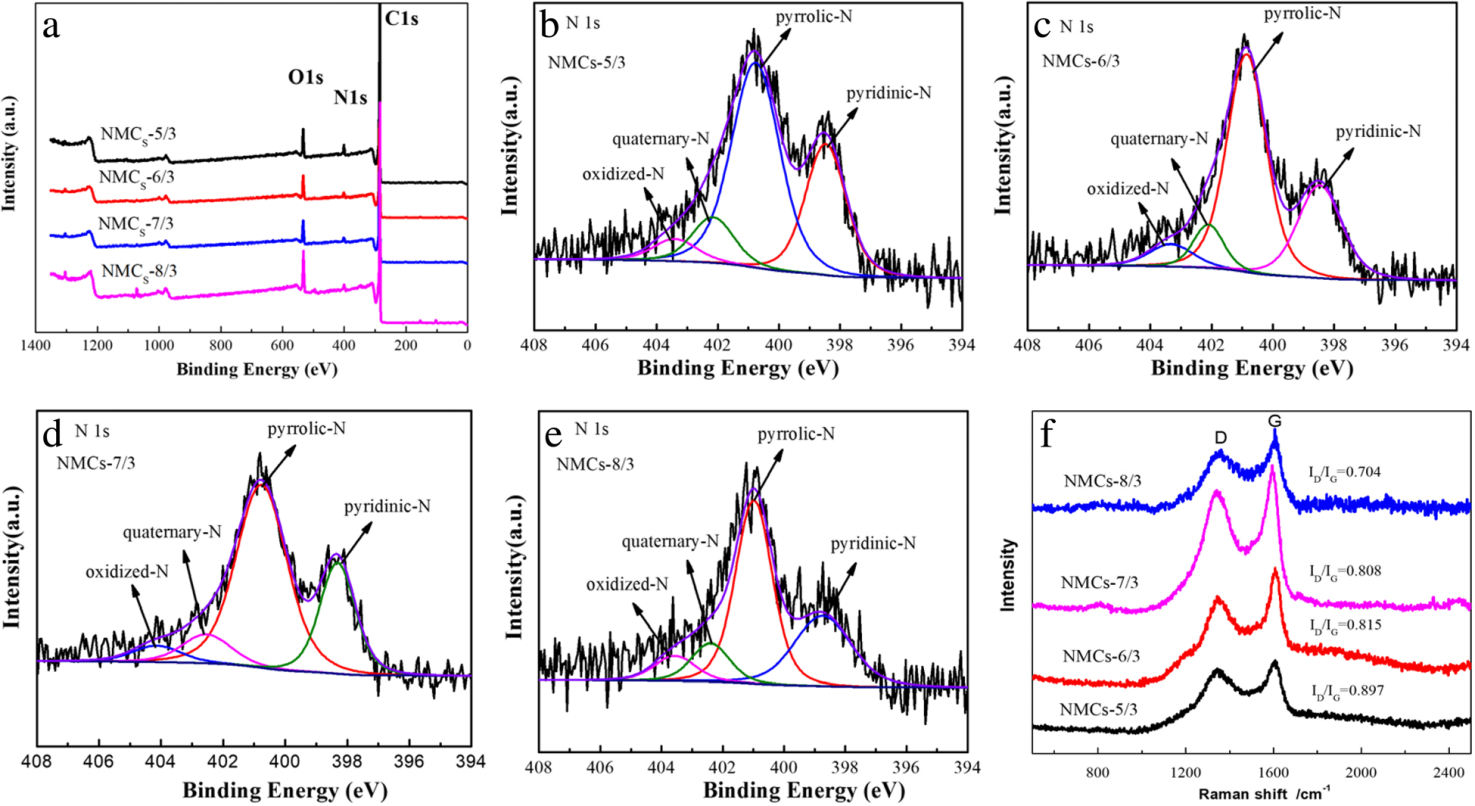
XPS survey of the NMCs (**a**) and N1s XPS spectra of NMCS-5/3 (**b**), NMCs-6/3 (**c**), NMCs-7/3 (**d**), NMCs-8/3 (**e**). Raman(**f**) patterns of the NMCs of the NMCs-*x*/3(*x* = 5, 6, 7, and 8)

The XPS graphs of the four NMCs are shown in Fig. 6 indicating that the four samples contained O, N, and C (Fig. 6a). N1s was processed with peak differentiation and fitting (Fig. 6b–e), showing a splitting of N1s into four peaks with corresponding binding energies of 398.37, 400.80, 402.40, and 404.53 eV, respectively, attributed to pyridinic nitrogen (N-6), pyrrolic nitrogen (N-5), quaternary nitrogen (N-Q), and oxidized –N (N–O) [36, 42–45]. Further, the peak areas of N-5 and N-6 were larger, indicating a greater content within the composite. These results clearly indicate that in situ *N* was doped into the mesoporous carbons, taking the form of pyridinic and pyrrolic nitrogen.

Table 2 shows the C, N, and O contents on the surface of the four NMCs obtained from XPS and elemental analyses (the differences in elemental content detected by each technique were subtle distinction). The total *N* content on the surface of the NMCs is greatest for NMCs-5/3, followed by NMCs-6/3, NMCs-7/3, and, finally, NMCs-8/3. Thus, as the C/Si ratio increase, the N content on the surface of NMCs gradually decreases. This phenomenon is attributed to the fact that, at lower C/Si ratios, the greater the amount of Si–OH in the system and the lower the amounts of –OH and –NH_2_ on CS available for hydrogen bonding. Therefore, there are more chances for –NH_2_ to contact with Si–OH strong in the formed three-dimensional net structure and the binding force between them is stronger, leading to a higher amount of N left in the carbon skeleton during the calcination process. However, at higher C/Si ratios, –OH and –NH_2_ on CS cannot form a three-dimensional network structure by hydrolysis with TEOS, and therefore, less N is retained following the calcination process due to volatilization into the N atmosphere.

**Table 2:** Elemental composition of the nitrogen-containing mesoporous carbon materials by elemental analysis and XPS

Sample name		Elemental analysis		XPS		
	C (wt%)	N (wt %)	H (wt %)	C (at.%)	N (at.%)	O (at.%)
NMCs-5/3	79.15	6.043	2.740	88.13	4.08	7.79
NMCs-6/3	76.26	5.710	2.888	84.32	3.42	12.25
NMCs-7/3	77.94	5.026	2.724	88.42	2.61	8.98
NMCs-8/3	78.23	4.753	2.562	86.99	2.39	10.62

Raman spectrum of carbon material NMCs-*x*/3 is shown in Fig. 6f. Two distinct characteristic peaks appeared in all samples at 1350 cm^−1^ and 1601 cm^−1^, corresponding to *D* and *G* peaks of carbon materials, respectively. Among them, *D* peak reflects the degree of atomic displacement, disordered carbon, edge defects, and other defects (sp^3^ carbon, dangling carbon, and vacancies, etc.) in carbon materials, and *G* peak reflects the degree of ordering of sp^2^ carbon. A ratio of *D* peak to *G* peak (*I*_*D*_/*I*_*G*_) can reflect the degree of crystallinity of carbon materials [46]. It is pointed out that the order of *I*_*D*_/*I*_*G*_ value is the same as that of *N* content on its surface and more defects are generated with the increasing nitrogen content [47]. The calculated results show that the *I*_*D*_/*I*_*G*_ of the four carbon materials, NMCs-5/3, NMCs-6/3, NMCs-7/3, and NMCs-8/3, are 0.897, 0.815, 0.808, and 0.704, respectively, and the order of their size is the same as that of their nitrogen content (see Table 2). It can be seen that the larger *I*_*D*_/*I*_*G*_ value of NMCs-5/3 indicates that the structural defect is more obvious, which is due to the large amount of nitrogen doping on the carbon material.

#### Hydrophilicity of NMCs

**Fig. 7:**
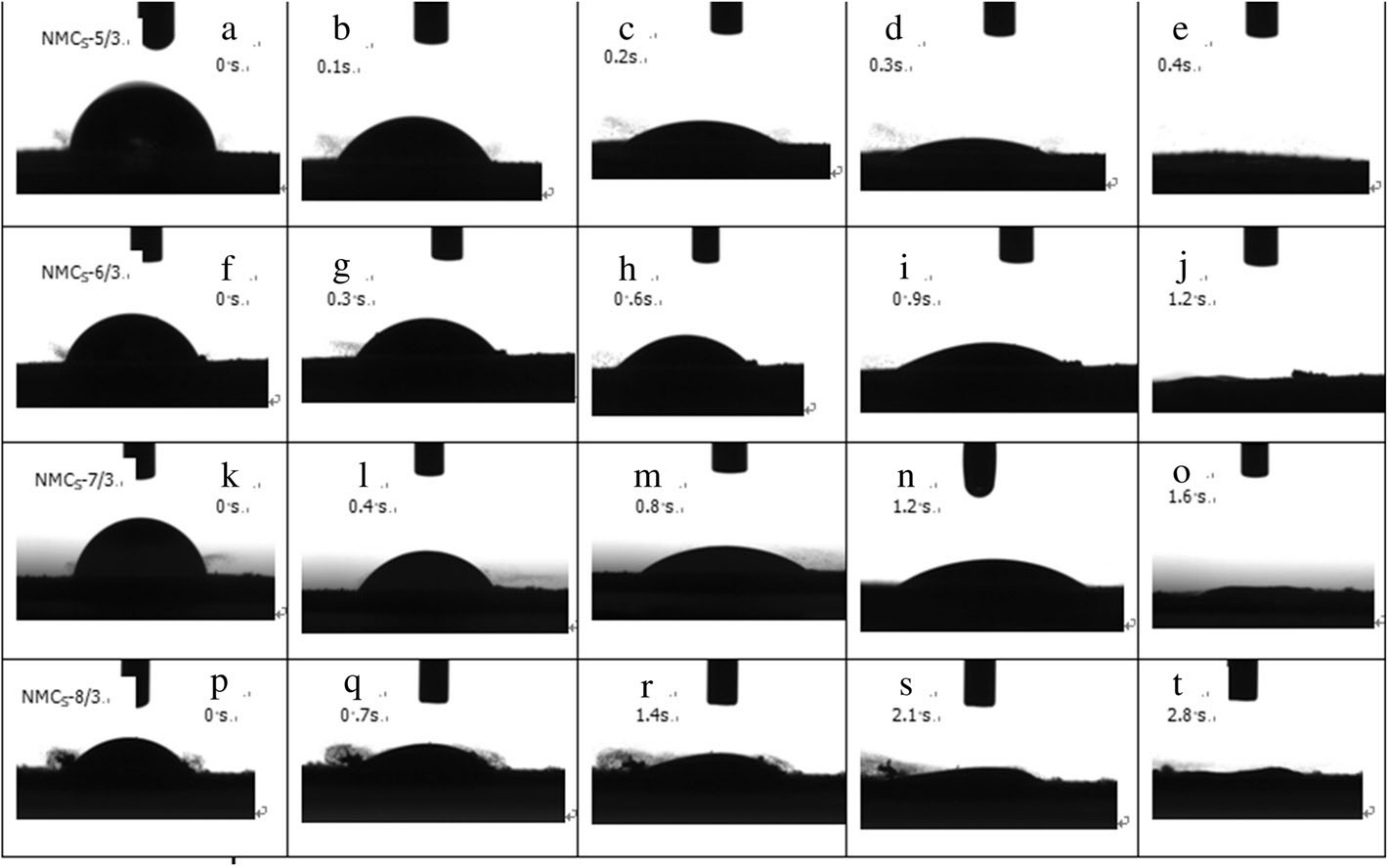
Optical micrographs of the water contact angles on the surface of mesoporous carbon as a function of contact time (**a**–**e**) NMCs-5/3, (**f**–**j**) NMCs-6/3, (**k**–**o**) NMCs-7/3, and (**p**–**t**) NMCs-8/3

The dynamic contact angles of water on NMCs-5/3, NMCs-6/3, NMCs-7/3, and NMCs-8/3 measured every 0.1 s (Fig. 7a–e), 0.3 s (Fig. 7f–j), 0.4 s (Fig. 7k–o), and 0.7 s (Fig. 7p–t) show that the required time to reduce the water droplet contact angle on NMCs-5/3, NMCs-6/3, NMCs-7/3, and NMCs-8/3 to below 20° was 0.45 s, 1.15 s, 1.54 s, and 2.71 s, respectively. Thus, the four samples show strong hydrophilicity compared to their non-nitrogen-doped mesoporous carbon counterparts (129°) [37]. The nitrogen elements in the NMCs formed active sites, leading to an increase in the sp^2^ cluster fraction, with an enhancement of surface roughness of the carbon materials [48], and thus, a smaller wetting angle and enhanced hydrophilicity and dispersibility. In addition, hydrogen bonding between N-5, N-6, and water molecules in NMCs also led to enhanced hydrophilicity [23, 49, 50]. The coupling of these effects allows for the potential application of NMCs in drug delivery.

**Fig. 8:**
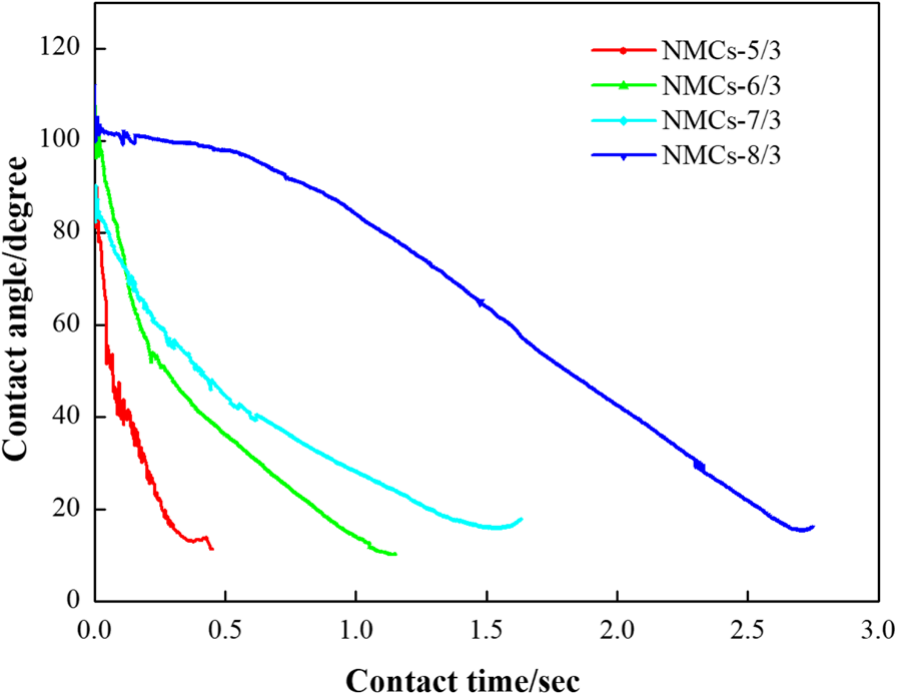
Plots of water contact angle on different mesoporous carbon versus contact time

Figure 8 is the relation curves of the variation of contact angles on these four NMCs over time. As is shown from Fig. 8, the required time for reducing the contact angles of water droplets on NMCs-5/3, NMCs-6/3, NMCs-7/3, and NMCs-8/3 to below 20° was 0.45 s, 1.15 s, 1.54 s, and 2.71 s, respectively. The shorter the required time for realizing the same contact angle is, the better the hydrophilicity of samples will be. Obviously, the hydrophilicity of each NMCs can be ranked in a descending order as NMCs-5/3 > NMCs-6/3 > NMCs-7/3 > NMCs-8/3, which is in line with that of the content of N on mesoporous carbons. In other words, the highest content of N on NMCs-5/3 means the best hydrophilicity. This can be attributed to the fact that the higher the content of N on mesoporous carbon material is, the greater the surface roughness will be; in addition, the higher content of N-5 and N-6 also leads to the enhanced hydrogen bonding between NMCs and water molecules; these two coupled effects enhanced the hydrophilicity of NMCs that can explain why the contact time was the shortest.

### Evaluation of Adsorption and Release Properties of NMCs for HCPT

**Fig. 9:**
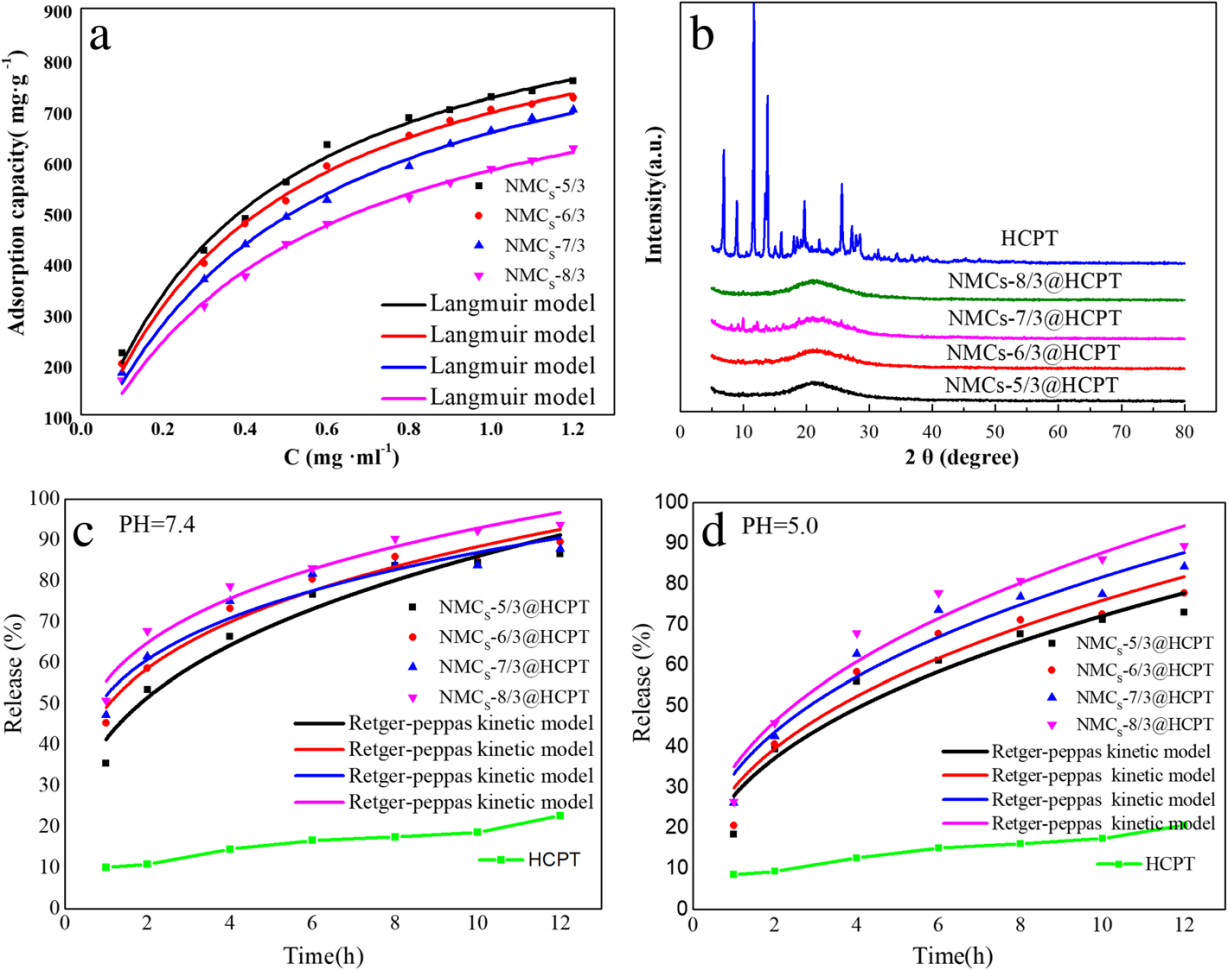
**a** HCPT adsorption isotherms of NMCs-*x*(*x* = 5, 6, 7, and 8) in ethanol solution. **b** The XRD patterns of pure HCPT and NMCs-*x*/3(*x* = 5, 6, 7, and 8)@HCPT. In vitro release profiles of HCPT from NMCS-*x*(*x* = 5, 6, 7, and 8)@HCPT and pure drug in pH = 7.4 (**c**) and pH = 5.0 (**d**) PBS solution

The HCPT adsorption curve of the four NMCs showed a gradually increasing adsorption capacity with increasing concentration of HCPT solution (Fig. 9a). This is attributed to the fact that the absorption and diffusion of HCPT in porous materials is based on the concentration gradient principle, wherein the higher the concentration of HCPT, the stronger the concentration gradient propulsion, and the greater the amount of HCPT arriving at the adsorption sites on the surface of NMCs for adsorptive preconcentration will be higher.

The experimental data retrieved from Fig. 9a was fitted using the Langmuir model (the processed data is provided in Table 3) using the Langmuir adsorption model equation, as follows:

$$ q={K}_L{q}_mc/\left(1+{K}_Lc\right) $$

**Table 3:** Langmuir constants of mesoporous carbons after drug loading with HCPT

Sample name	*q*_*m*/_mg g^−1^	*K*_*L*_/mg g min^−1^	*R*^2^
NMCs-5/3	1013.51	2.494	0.9929
NMCs-6/3	995.19	2.309	0.9967
NMCs-7/3	989.57	1.959	0.9956
NMCs-8/3	885.63	1.908	0.9937

where *q* is the mass of HCPT adsorbed in the porous structure per unit mass of NMCs at the equilibrium state(mg g^−1^), *q*_*m*_ is the saturated adsorption capacity of NMCs for HCPT(mg g^−1^), *c* is the concentration of HCPT at the equilibrium state of adsorption (mg mL^−1^), and *K*_*L*_ is the Langmuir adsorption constant (mg g min^−1^).

The adsorption of HCPT molecules in the porous structure of NMCs followed the Langmuir’s adsorption law. Additionally, the value of the absorption constant did not vary significantly, suggesting that the affinity for HCPT was similar among the four NMCs. Notably, the absorption capacity of all four NMCs for HCPT is higher, up to 1013.51 mg g^−1^ (50.33% drug loading), which is much higher than that of the non-N-doped three-dimensional macroporous carbon material (24% drug loading) for HCPT [51]. However, the absorption capacity of the four NMCs for HCPT is higher for NMC-5/3, followed by NMC-6/3, NMC-7/3, and, finally, NMC-8/3, in line with the order of the content of N on the surface of mesoporous carbons. Thus, the higher the N content on the surface of NMCs, the stronger its absorption capacity for HCPT. This could be attributed to the increased surface roughness and hydrophilicity enhancing the absorption capacity for HCPT.

The XRD patterns of pure HCPT and the mesoporous carbon adsorbed on HCPT NMCs-*x*/3 (*x* = 5, 6, 7, and 8)@HCPT are shown in Fig. 9b. Pure HCPT has a strong crystal diffraction peaks at 2*θ* = 6.9°, 9.0°, 11.70°, 13.86°, 19.73°, 25.65°, 27.27°, 27.91°, and 28.52°. It indicates that pure HCPT existed in the crystalline state. But when HCPT is loaded on mesoporous carbon, no diffraction peaks of HCPT are detected in NMCs-*x*/3 (*x* = 5, 6, 7, and 8)@HCPT samples. It means that HCPT adsorbed in mesoporous carbon is in an amorphous state, which is consistent with Qinfu Zhao’s report [5], the nanoporous channels of mesoporous carbon can make the drug in an amorphous and amorphous state, which is conducive to improving the drug dissolution rate.

The in vitro drug release behavior of HCPT in the NMCs and of pure drug HCPT in PBS (pH 7.4 and 5.0) was assessed (Fig. 9c, d). The pure drug release rate into PBS after 1 h is only 9.96% and increase to 22.7% in 12 h. In contrast, the drug release rate is significantly improved when HCPT drug molecules are absorbed onto the four NCMs, showing a drug release rate of 35.42~50.80% and 86.67~93.75% at 1 and 12 h, respectively. Similar results are obtained in Fig. 9d in phosphate buffer solution (pH = 5.0). These observations are attributed to the fact that the nanoporous structure of mesoporous carbon inhibits drug crystallization (see Fig. 9b), leading to drug absorption in the microcrystalline or amorphous state, and thereby increasing its solubility and release rate [52].

The experimental data retrieved from Fig. 9c, d were fitted using a Retger-Peppas kinetic equation (the processed data is provided in Table 4), as follows:

$$ Q={kt}^n $$

**Table 4:** Parameters obtained by fitting of Retger-Peppas kinetic equation

	Sample name	*k*	*n*	*R*^2^
pH = 7.4	NMCs-5/3@HCPT	41.321	0.3186	0.9498
	NMCs-6/3@HCPT	49.094	0.2552	0.9615
	NMCs-7/3@HCPT	52.081	0.2224	0.9308
	NMCs-8/3@HCPT	55.569	0.2230	0.9565
pH = 5.0	NMCs-5/3@HCPT	27.908	0.4123	0.9114
	NMCs-6/3@HCPT	29.808	0.4059	0.9113
	NMCs-7/3@HCPT	33.245	0.3903	0.9294
	NMCs-8/3@HCPT	34.994	0.3988	0.9305

where *Q* is the fractional release of HCPT, *t* is the time of release, and *k* and *n* are the release rate constant and index, respectively.

It can be seen from the figures and tables that the *k* value of the drug release rate is closely related to the nitrogen content of mesoporous carbon materials. NMCs-5/3 with the highest nitrogen content (6.043%) exhibits the slowest release rate (*k* value is smaller), while NMCs-8/3 with the lowest nitrogen content (4.753%) exhibits the fastest release rate (*k* value is larger). This may be attributed to the fact that the high nitrogen content mesoporous carbon material NMCs-5/3 has more active sites than the low nitrogen content mesoporous carbon material NMCs-8/3, thus showing a stronger interaction with HCPT, and its hindered diffusion and release into the medium.

The release rate of HCPT in an acidic environment with pH 5.0 is slower than that in a neutral environment with pH 7.4. It can be seen that the release rate of HCPT is pH dependence, and the slower the release rate is in the environment with lower pH value. Because the microenvironments of extracellular tissues and intracellular lysosomes and nucleosomes of tumors are acidic [12], slow release of HCPT from phosphate buffer solution at pH = 5.0 in an acidic environment can achieve the goal of long-term anti-tumor.

Thus, mesoporous carbon has a high nitrogen content and good hydrophilicity, and it has a large adsorption capacity for anti-cancer drug HCPT. At the same time, high nitrogen content increases the adsorption of HCPT and reduces the release rate of drugs from mesoporous channels. The more nitrogen content of mesoporous carbon is, the slower drug release is; on the contrary, the lower nitrogen content of mesoporous carbon is, the faster drug release. Therefore, the release rate of HCPT can be controlled by adjusting the nitrogen content and pH value of mesoporous carbon materials.

## Conclusion

Nanospherical mesoporous carbon materials are successfully prepared with high specific surface area (1342.9–2061.6 m^2^/g), narrowly pore size distribution (2.01–3.65 nm), and high nitrogen content (4.75–6.04%). As the C/Si ratio increased, the specific surface area and the mesopore volume of NMCs first increased and then decreased, and when C/Si ratio is 7:3, the NMC-7/3 has the largest, *S*_BET_ (2061.6 m^2^/g) and *V*_Mes_ (0.77 cm^3^/g), and higher N content (5.026%). The doping of in situ N increases the hydrophilicity of NMCs, which increased gradually with the surface N content. NMC-5/3 has the highest N content along with the best hydrophilicity.

All four NMCs show a good adsorption capacity for the antitumor drug HCPT. The absorption capacity of NMCs-*x* towards HCPT is in the following orders: *q*_NMCs-5/3_ > *q*_NMCs-6/3_ > *q*_NMCs-7/3_ > *q*_NMCs-8/3_, which is consistent with the order of N content on the material surface, and NMCs-5/3 has the largest saturated adsorption capacity of HCPT (1013.51 mg g^−1^), and higher dissolution rate (93.75%). NMCs loaded with HCPT significantly increase the drug release rate. Moreover, the higher the nitrogen content of the mesoporous carbon material, the lower the release rate of the drug HCPT due to more active sites, and the release rate in the neutral environment of pH = 7.4 was higher than that in the acidic environment of pH = 5.0. Thus, the NMCs show potential drug delivery applications for water-insoluble antitumor drugs.

## Data Availability

All datasets are presented in the main paper or in the additional supporting files.

## Abbreviations

BET: Brunauer-Emmett-Teller
C/Si: Carbon-to-silicon
CS: Chitosan
FTIR: Fourier Transform infrared spectroscopy
HCPT: Hydroxycamptothecin
–NH_2_: Amino
NMCs: Nitrogen-doped mesoporous carbon spheres
–OH: Hydroxyl
PBS: Phosphate buffer solution
Raman: Raman spectra
SEM: Scanning electron microscope
Si–OH: Silicon hydroxyl
TEM: Transmission electron microscopy
TEOS: Tetraethyl orthosilicate
TG: Thermogravimetry
XPS: X-ray photoelectron spectroscopy
XRD: X-ray powder diffraction
